# The LRRK2 N-terminal domain influences vesicle trafficking: impact of the E193K variant

**DOI:** 10.1038/s41598-020-60834-5

**Published:** 2020-03-02

**Authors:** Algerta Marku, Maria Dolores Perez Carrion, Francesca Pischedda, Antonella Marte, Zeila Casiraghi, Paola Marciani, Felix von Zweydorf, Christian Johannes Gloeckner, Franco Onofri, Carla Perego, Giovanni Piccoli

**Affiliations:** 1 Department of Excellence of Pharmacological and Biomolecular Sciences Università degli Studi di Milano, Milano, Italy; 2 CIBIO, Università degli Studi di Trento, Italy & Dulbecco Telethon Institute, Trento, Italy; 3 Facultad de Farmacia, Universidad CEU San Pablo, Madrid, Spain; 4 Department of Experimental Medicine, University of Genova, Genova, Italy; 5 German Center for Neurodegenerative Diseases (DZNE), Tübingen, Germany; 6 University of Tübingen, Center for Ophthalmology, Institute for Ophthalmic Research, 72076 Tübingen, Germany; 7 IRCCS Ospedale Policlinico San Martino, 16132 Genova, Italy

**Keywords:** Cell biology, Neuroscience

## Abstract

The LRRK2 protein consists of multiple functional domains, including protein-binding domains at its N and C-terminus. Mutations in the Leucine-rich repeat kinase 2 gene (*LRRK2*) have been linked to familial and sporadic Parkinson’s disease (PD). We have recently described a novel variant falling within the N-terminal armadillo repeats, E193K. Herein, our aim is to investigate the functional impact of LRRK2 N-terminal domain and the E193K variant on vesicle trafficking. By combining Total Internal Reflection Fluorescence (TIRF) microscopy and a synaptopHluorin assay, we found that expression of a construct lacking the N-terminal domain increases the frequency and amplitude of spontaneous synaptic events. Complementary biochemical approaches showed that the E193K variant alters the binding properties of LRRK2, decreases LRRK2 binding to synaptic vesicles, and promotes vesicle fusion. Our results confirm the physiological and pathological relevance of the nature of the LRRK2-associated macro-molecular complex solidifying the idea that different pathological mutations critically alter the scaffolding function of LRRK2 resulting in a perturbation of the vesicular trafficking as a common denominator.

## Introduction

Parkinson’s disease (PD) is the second most common age-related neurodegenerative disorder. PD affects 2% of the population above 65-years and is clinically characterized by movement impairments such as bradykinesia, rigidity and resting tremor. PD is related to the accumulation of protein aggregates (Lewy bodies) and loss of dopaminergic neurons in the *substantia nigra*^1^. The majority of PD cases do not correlate with clear genetic causes, nonetheless, late-onset autosomal dominant PD have been robustly linked to mutations in the Leucine-rich repeat kinase 2 *(LRRK2)* gene (PARK8; OMIM 609007). Mutations in *LRRK2* account for up to 13% of familial PD cases and have also been identified in 1 to 2% of idiopathic PD patients^2^. LRRK2 is a complex protein that consists of multiple functional domains, allowing a dual enzymatic activity and multiple protein-protein interactions^3^. We have contributed to describe LRRK2 as a synaptic protein that influences the synaptic vesicle (SV) cycle^4–7^. Noteworthy, LRRK2 rodent models show age-related neurotransmission defects^8–10^ and LRRK2 binds and phosphorylates several presynaptic proteins^11–14^. The identification of LRRK2 as a PD causative gene boosted the interest on its GTPase and kinase domains, and the possible link of the modified enzymatic activities to the pathological cascade^15^. However, genetic studies have identified pathological relevant variants in domains outside the catalytic core. In Chinese Han and Korean ethnicity, increased risk of developing idiopathic PD is associated with the G2385R variant within the C-terminal WD40 domain^16,17^. Recently, we investigated the functional impact of the E193K variant identified in an Italian family with 3 siblings affected by PD^18^. E193K falls within the N-terminal armadillo repeat structure. LRRK2 N-terminal Armadillo, the C-terminal WD40 domain, LRR and Ankyrin repeats act as a hub orchestrating various protein interactions^3^. In this study, we demonstrated that E193K variant modifies the LRRK2-protein interactome and lessens its binding to SV. We combined TIRF microscopy and a synaptopHluorin assay and found that LRRK2-E193K expression increases the frequency and time length of SV fusion events.

## Results

### Impact of LRRK2 N-terminal domain on vesicle trafficking

**Figure 1:**
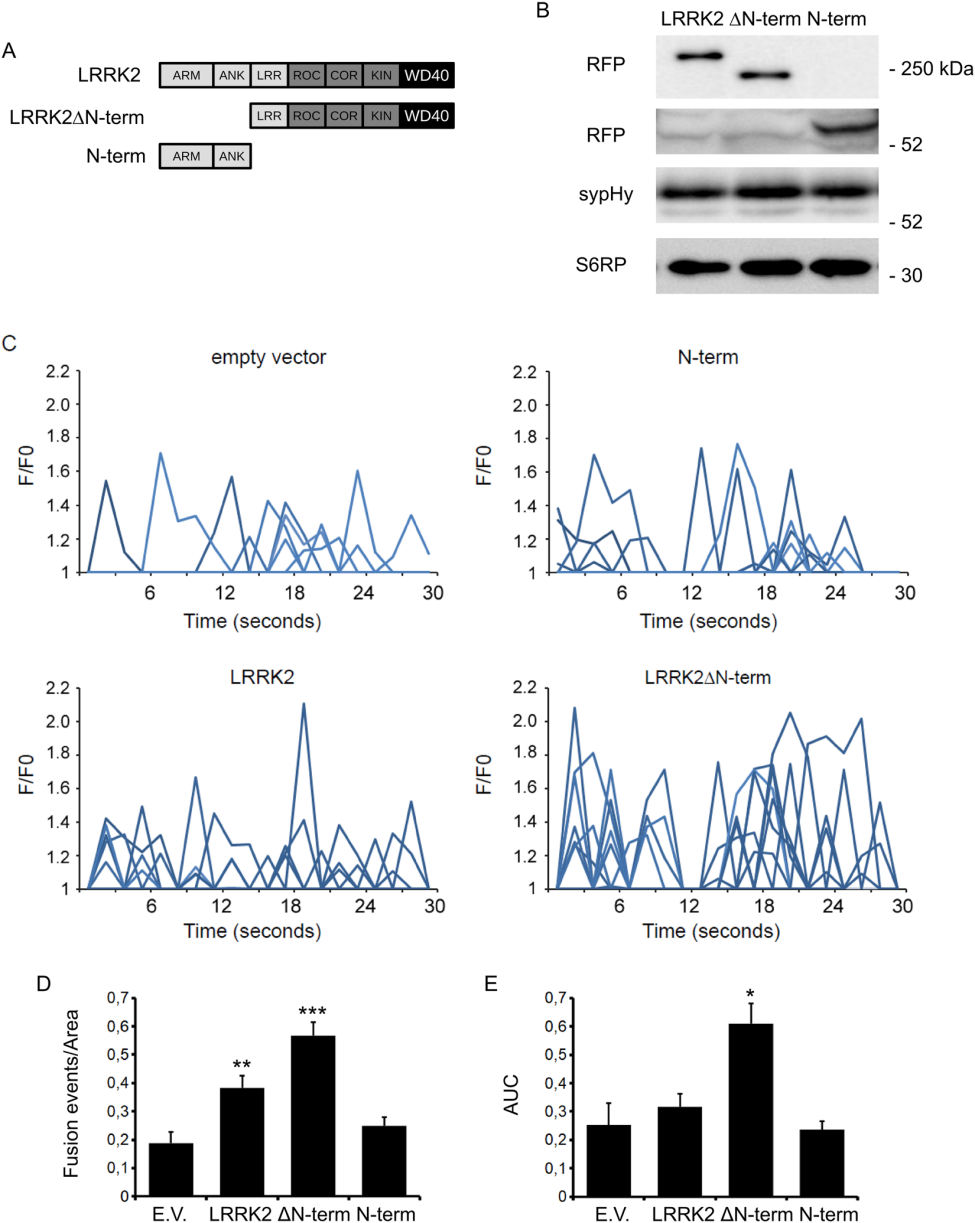
Domain-wise dissection of LRRK2 impact on vesicle trafficking. (**A**) Schematic representation of RFP-LRRK2 derived constructs. The distinct LRRK2 domains are indicated. Protein-protein domains: ARM, armadillo repeats; ANK, ankyrin repeats; LRRs, leucine-rich repeats; WD40, WD40 repeats; Roc, Ras of complex proteins; COR, C-terminal of ROC; Kin, kinase domain. (**B**) Western blotting analysis of cells expressing synaptopHluorin reporter (sypHy) together with RFP-LRRK2 derived constructs. (**C**) Time course analysis of fusion events occurring in N2A cells transfected with the different LRRK2 derived constructs. N2A cells were co-transfected with sypHy reporter and empty vector (E.V.) or the indicated RFP-LRRK2 derived constructs. TIRFM imaging was performed 48 h after transfection. Peaks of fluorescence intensity correspond to single fusion events. Fluorescence data are expressed as F/F0. The graphs show the total number of fusion events (**D**) and the resulting fluorescence changes (**E**) expressed as Area Under Curve (AUC) for each construct. Data are normalized for the cell area and are expressed as mean ± SE; n = 20 cells per construct, in three independent experiments. *p < 0.05, **p < 0.01, ***p < 0.001 versus empty vector, ANOVA.

Since the N-terminal Armadillo domain is involved in LRRK2 supra-molecular organization^19^, we further investigate the functional role of LRRK2 N-terminal domain by means of TIRF microscopy (TIRFM) coupled with a synaptopHluorin assay as previously described^20^. For this purpose, we over-expressed in N2A neuronal line the sypHy reporter together with a panel of RFP-tagged LRRK2 derived constructs: LRRK2 full-length, LRRK2 lacking the first 913 amino acids (hereinafter LRRK2ΔN-term) as well as a complementary a deletion construct LRRK2 aa 1–983, containing the N-terminal Armadillo and Ankyrin domains (hereinafter N-terminal domain) (Fig. 1A). First of all, by western-blotting analysis we verified that the different LRRK2 constructs show similar expression yield and do not overtly influence the sypHy level (Fig. 1B and Supplementary Fig. 1A,B). Next, we analysed SV dynamics by TIRFM. Upon over-expression, full-length wild-type LRRK2 significantly increased the number of spontaneous fusion events (Fig. 1D,E) without changing single peak intensities (Fig. 1C and Supplementary Fig. 1E). The expression of the isolated LRRK2 N-terminal domain did not elicit SV dynamics as judged by the quantification of number of events, total fluorescence intensity (Fig. 1C–E) or increase in peak fluorescence intensity (Supplementary Fig. 1E). Interestingly, the expression of LRRK2 ΔN-term enhanced the frequency of fusion events and total fluorescence elicited (Fig. 1C–E and Supplementary Fig. 1E). Altogether, our findings indicate that the N-terminal domain (i.e. containing the Armadillo and Ankyrin domains) is a critical effector for the LRRK2 function in the SV dynamics.

### The N-terminal domain influences LRRK2 interactions

**Figure 2:**
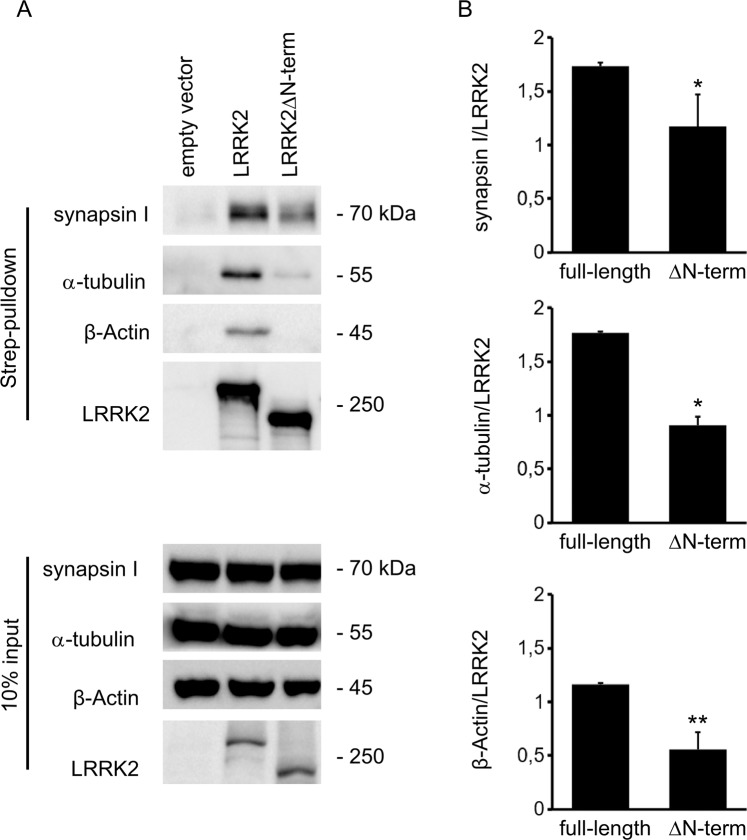
LRRK2 interacts with proteins involved in vesicle trafficking. (**A**) We isolated on streptavidin resin full-length Strep-FLAG-LRRK2 and Strep-FLAG-LRRK2ΔN-terminus proteins from N2A over-expressing cells. Interacting proteins were resolved by western-blotting. (**B**) We evaluated the extent of Synapsin I, α-tubulin and β-Actin bound to the different LRRK2 variants. Data are expressed as optical density and normalized versus amount of precipitated LRRK2 protein. Graphs report mean ± SE, *p < 0.05, **p < 0.01 Student’s T-test, n = 4 independent experiments.

We have brought evidence that LRRK2 modulates SV trafficking via interaction with synapsin I, α-tubulin and β-actin^4–6^. To appreciate the role of the LRRK2 N-terminal domain in such interactions, we expressed Strep-FLAG full-length LRRK2 and Strep-FLAG LRRK2ΔN-term in N2A cells. Upon streptavidin-pull-down, we analysed interacting proteins by western-blotting (Fig. 2A). Interestingly, we found that the interaction with synapsin I, α-tubulin and β-actin was greatly decreased in the LRRK2ΔN-term variant (Fig. 2B). In conclusion, our data suggest that the N-terminal domain influences LRRK2 interactions.

### The E193K variant affects vesicle trafficking

**Figure 3:**
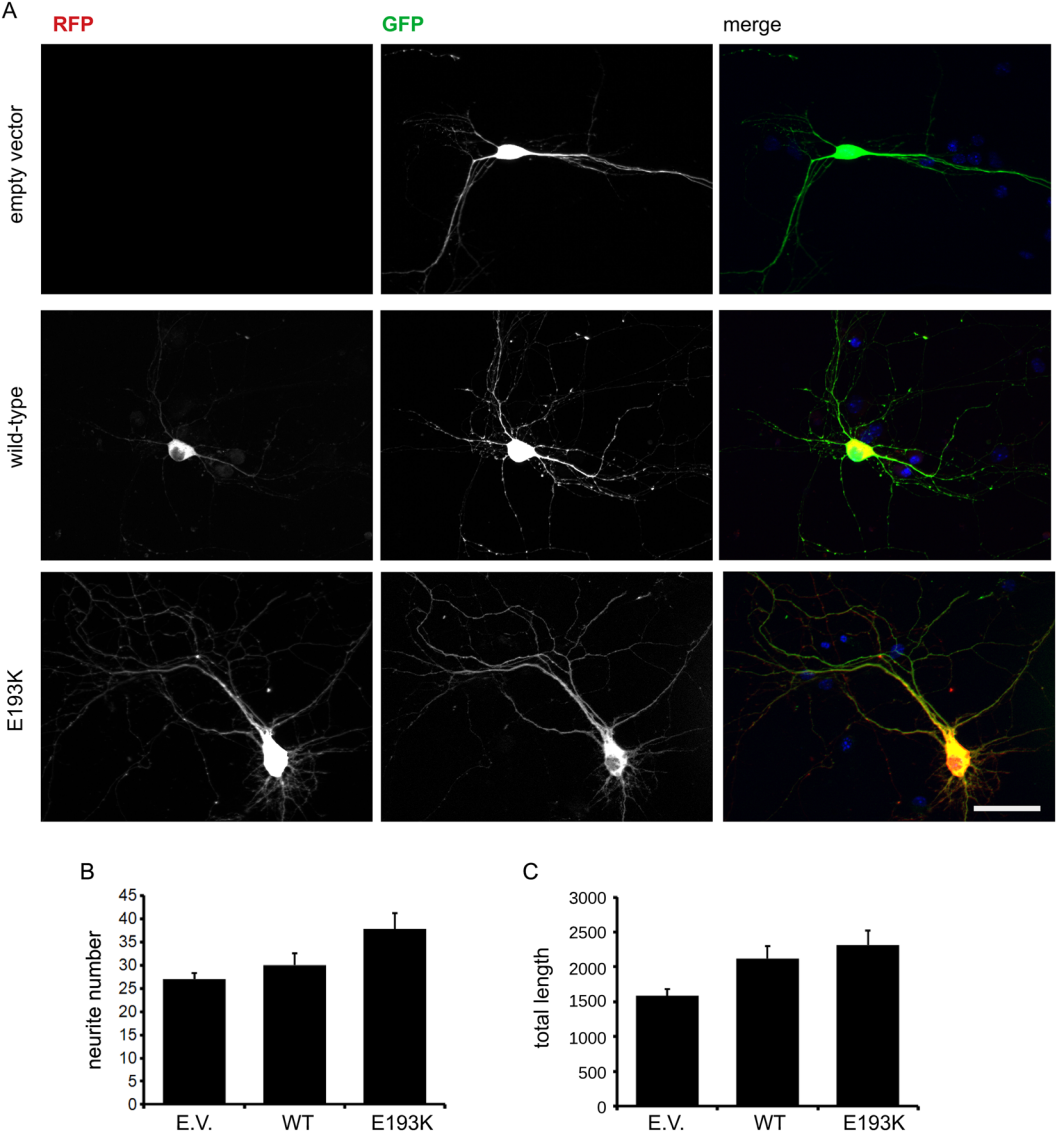
E193K variant does not influence neuritic tree. (**A**) We expressed in DIV4 cortical neurons GFP together with empty vector or RFP-LRRK2 wild type or RFP-LRRK2 E193K variant. We processed culture at DIV14 for imaging purposes. Scale bar = 50 μm. (**B**) The graph reports neurite number, expressed as mean ± SE, n = 12 cells. (**C**) The graph reports total neurite length, expressed as mean ± SE, n = 12 cells.

**Figure 4:**
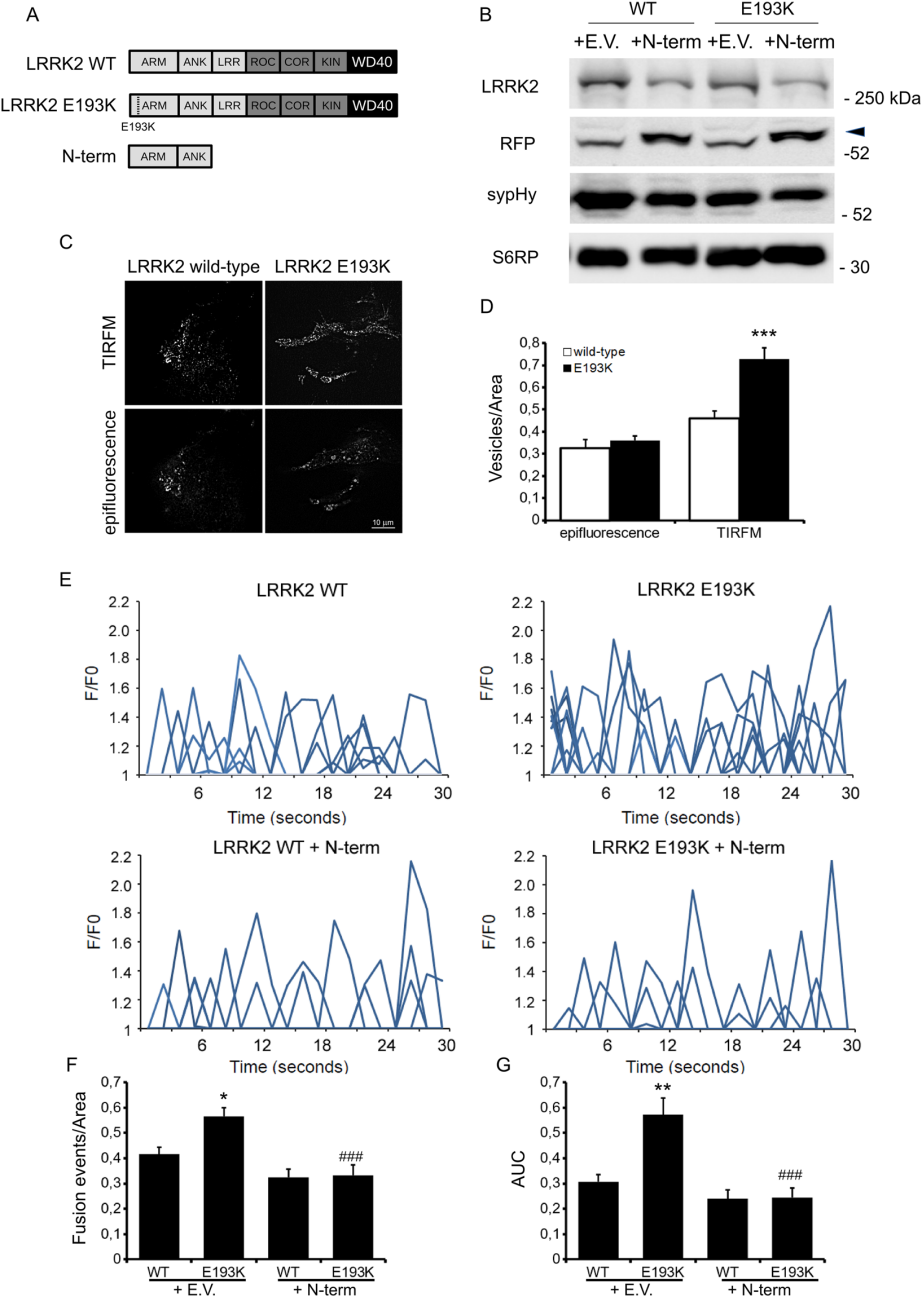
E193K variant affects vesicle trafficking. (**A**) Schematic representation of LRRK2 wild type, E193K variant and N-terminus domain. The distinct LRRK2 domains are indicated. (**B**) Western-blotting analysis of WT and E193K N2A clones expressing sypHy reporter together with empty vector or RFP-LRRK2 N-terminus constructs. Arrowhead indicates the specific RFP positive band detected by the anti-RFP antibody. (**C**) Vesicle density in the TIRFM zone after NH_4_Cl treatment. The cells were incubated with the membrane permeant NH_4_Cl solution for 5 minutes, to label all sypHy positive clusters. Then, cells were fixed and imaged by epifluorescence or TIRFM to visualize vesicles docked to the plasma membrane. Each spot corresponds to a sypHy positive cluster. Scale bar = 10 μm. (**D**) The graph reports the number of sypHy positive clusters visualized in the same cell under epifluorescence or TIRFM. Data are normalized for the cell area and are expressed as mean ± SE; n = 15 cells for construct. ***p < 0.001, Student’s T-test. (**E**) Time course analysis of synaptic events occurring in N2A stable clones expressing LRRK2 wild type (WT) or E193K LRRK2 and transfected with the empty-vector or with the isolated N-terminal domain together with the sypHy reporter. Transfected cells were imaged by TIRFM 48 h later. Peaks of variable fluorescence intensity correspond to single fusion events. Fluorescence data are expressed as F/F0. The graphs show the total number of fusion events (**F**) and the resulting fluorescence changes (**G**) expressed as Area Under Curve (AUC) for each construct. Data are normalized for the cell area and are expressed as mean ± SE of up to 20 cells per construct, in three independent experiments. *p < 0.05, **p < 0.01 compared to LRRK2 wild type, ^###^p < 0.001 compared to empty vector transfected clones.

Our previous *in silico* and *in vitro* analyses demonstrated that the E193K variant affects deeply the properties of LRRK2 Armadillo domain^18^. Several reports describe that different LRRK2 mutations influence neural morphological maturation^21^. Thus, we transfected a GFP reporter either together with an empty vector or RFP-LRRK2 wild-type or RFP-LRRK2 E193K in DIV4 cortical neurons. On DIV14, we set up cultures for imaging analysis of the neuritic tree (Fig. 3A). We noticed that the expression of LRRK2 wild-type or the E193K variant did not have a major effect, neither the neurite number nor their total length (Fig. 3B,C). Although our experimental strategy may have missed transient effect happening during neuronal development, our data suggest that E193K has no major impact on mature neuritic tree. The common kinase-activating G2019S mutation affects SV trafficking in cortical neurons^11,14^ as well as upon over-expression in N2A cells (Supplementary Fig. 2). Thus, we addressed our studies to the impact of the E193K variant on SV trafficking. We analysed the trafficking of sypHy vesicles by TIRFM using an N2A line stably expressing either wild-type LRRK2 or the E193K variant (Fig. 4A). By western blotting analysis we found that the level of expression of LRRK2-derived constructs were comparable to each other and the sypHy reporter level was not significantly affected (Fig. 4B and Supplementary Fig. 1C,D). First, we analysed whether the LRRK2 E193K variant might affect SV number or intracellular distribution. To this aim, we manifested the total pool of sypHy vesicles *via* NH_4_Cl basification. Under epifluorescence microscopy cells expressing wild type or LKKR2 did not count overt different number of vesicles. Instead, once we focused our analysis to the TIRF zone (i.e. in a range of up to 100 nm away from the plasma membrane) we detected significantly more sypHy clusters in cells expressing LRRK2 E193K (Fig. 4C,D). Interestingly, as we monitored SV dynamic by TIRFM, we found an increase in the number of fusion events upon LRRK2 E193K expression (Fig. 4E–G and Supplementary Fig. 1F). This indicates that the E193K variant might abolish the physiological function of the Armadillo domain. Therefore, we attempted to rescue E193K phenotype on vesicle trafficking by co-expressing the isolated N-terminal domain. The co-expression of the LRRK2 N-terminus abolished the increase in the frequency of fusion events and the associated change in total fluorescence detectable upon LRRK2 E193K expression (Fig. 4E–G and Supplementary Fig. 1F). Collectively, these findings confirm the key role of the N-terminal domain in the contest of SV dynamics and support the pathological relevance of the LRRK2 E193K variant.

### The E193K variant alters LRRK2-binding properties

**Figure 5:**
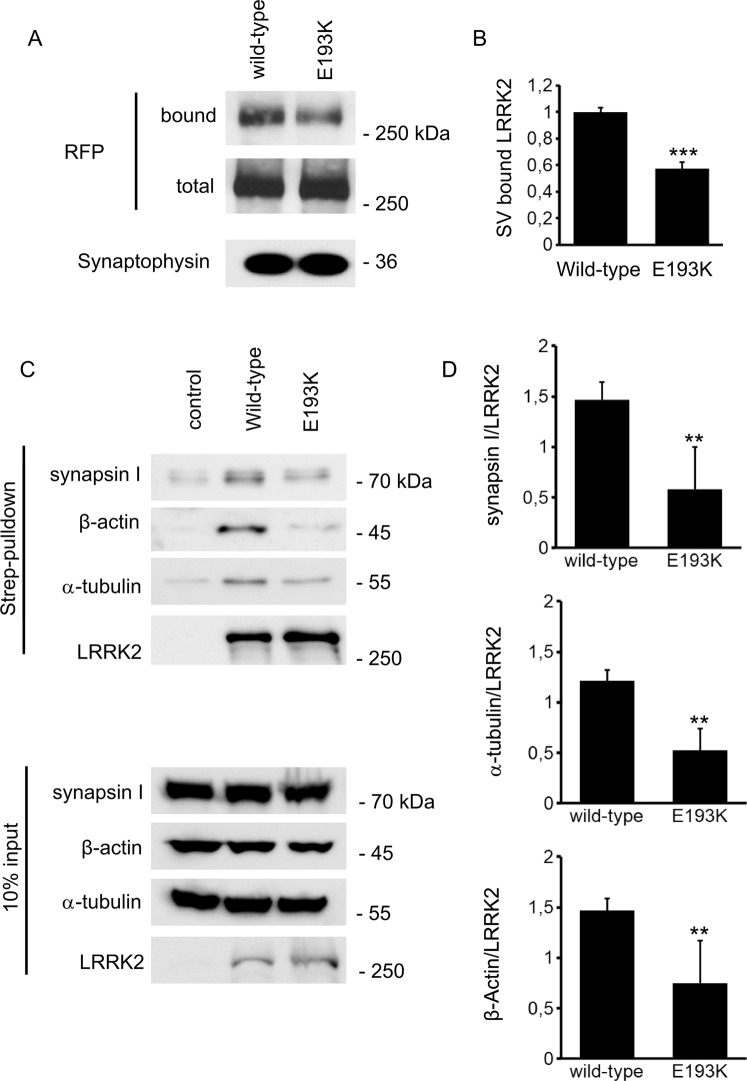
E193K mutation affects LRRK2 binding properties. (**A**) We measured the extent of LRRK2 and SV binding by ultracentrifugation sedimentation assay. We incubated purified RFP-LRRK2 wild type and E193K variant with isolated SV (10 μg protein/sample). Bound RFP-LRRK2 was separated from free RFP-LRRK2 by high-speed centrifugation. We appreciated SV-bound LRRK2 by immunoblotting with anti-RFP antibody. The recovery of SV in the pellet was evaluated based on synaptophysin immunoreactivity. (**B**) The binding of RFP-LRRK2 wild type and E193K to SV was calculated as the ratio of total RFP-LRRK2 and expressed as mean ± SE, n = 6, ***p < 0.001, Student’s t-test. (**C**) We isolated on streptavidin resin full-length FLAG-LRRK2 wild-type and FLAG-LRRK2 E193K proteins from N2A over-expressing cells. Interacting proteins were resolved by western-blotting. (**D**) We evaluated the extent of synapsin I, α-tubulin, β-Actin bound to the different LRRK2 variant. Data are expressed as the ratio over LRRK2 wild-type. Graphs report mean ± SE; n = 4. **p < 0.01, Student’s T-test.

We have already described that the E193K variant alters biochemical properties of LRRK2^18^. To investigate whether the E193K variant perturbs the interaction between LRRK2 and SV, we incubated either full-length LRRK2 wild-type or E193K RFP-fusion protein with purified SV at a nanomolar concentration and tested the magnitude of binding *via* a high-speed sedimentation assay. Taking advantage of a RFP-specific antibody, western blotting analysis showed that LRRK2 E193K binds less efficiently to purified SV than LRRK2 wild type (Fig. 5A,B). Since the stability of LRRK2 binding to SV might be affected by differences in terms of interacting proteins, we focused on the binding properties of LRRK2 wild-type and E193K variants. First, we purified Strep-FLAG-tagged full length wild-type LRRK2 and the E193K variant after recombinant expression in the N2A cell line. Subsequently we resolved the eluted proteins by western blotting. Our results showed that the E193K variant reduces affinity of LRRK2 towards a collection of selected proteins, i.e. synapsin I, α-tubulin and β-actin (Fig. 5C,D). Altogether, our results indicate that the E193K substitution induces structural modifications that alter LRRK2 binding properties.

## Discussion

Accumulating evidence is raising the interest on LRRK2 functions other than kinase activity. In particular, the community is gaining deep insight into the functional role of the LRRK2 interactome^22–24^. LRRK2 encompasses several domains involved in protein-protein interactions, namely the armadillo and LRR repeats at the N-terminus as well as WD40 repeats at the C-terminus^3^. Furthermore, it has been shown that the armadillo repeats mediate the interaction between LRRK2 and FADD, which is critical to elicit LRRK2-related toxicity^25^. Our recent characterization of the E193K variant within the armadillo repeats stresses out the physiological relevance of LRRK2 N-terminal domain. By combining TIRFM with a synaptopHluorin assay, here, we resolved the functional implication of N-terminal LRRK2 domain in the context of SV fusion. We reported that the over-expression of a construct expressing a LRRK2 fragment, missing the N-terminal domain, increases the number of fusion events. These observations bring further support to the model describing LRRK2 as a key player in SV dynamics^5^. We deem that this function relies on LRRK2 binding to proteins involved in SV trafficking such as tubulin, actin, synapsin but also directly to SV. Indeed, our previous works pinpointed the WD40 domain as the key domain allowing LRRK2 interaction with SV-associated proteins and SV themselves. In particular, we showed that the isolated WD40 domain binds directly to SV and hosts the interactions with α-tubulin, β-actin and synapsin I^6^. At a first glance, those data are in contradiction with our new observation that the N-terminal armadillo domain affects the interaction between LRRK2 and SV-associated proteins, as well. Accumulating literature describes LRRK2 as a dimer characterized by a complex architecture where distant domains are engaged in multiple contacts^19,26^. In particular, Guitoli *et al*. combined homology models, molecular docking and experimental constraints to generate a model of the dimeric LRRK2 holoenzyme where the C-terminal WD40 domain is in close proximity to the N-terminal domains^19^. Our data are consistent with such model and suggest that the N-terminal and C-terminal domains act together to shape the interacting site for SV and SV-associated proteins. However, we reported that the isolated LRRK2-WD40 domain prevents SV fusion by itself^4,6^. In contrast, the isolated N-terminal fragment did not elicit an overt effect on SV dynamics. Such different outcome might arise from the intrinsic nature of the two domains: while WD40 repeats are evolutionary conserved domains allowing interaction with a large variety of biomolecules, such as DNA, proteins and cellular membranes^27–29^ Armadillo domains have mainly been associated to cytoskeletal structures^30^. This outcome may be explained by a model where the N-terminal armadillo domain contributes to organize LRRK2 dimer. If this holds true, an alteration in the N- terminal domain might impact on LRRK2 dimer organization and eventually on LRRK2 biological function, including SV dynamics. Alternatively, the N-terminal domain might act as a molecular inhibitor dictating LRRK2 interaction or intracellular localization. *In silico* prediction as well as *in vitro* evidence suggest that E193K variant impact on N-terminal domain folding and therefore might alter its biochemical features. In fact, E193K variant changes LRRK2 subcellular distribution, impairs 14-3-3 binding and influences LRRK2 binding features^18^. Eventually, such biochemical alterations can influence one established LRRK2 function, i.e. vesicle fusion. We observed that the E193K variant increases SV fusion. Interestingly, we and other have reported a similar phenotype upon LRRK2 silencing^4,5,31^. Furthermore, we were able to rescue the SV phenotype correlated with E193K upon co-expression of the isolated N-terminal domain. In the light of the current LRRK2 dimer models, the isolated N-terminal domain may support the organization of a functional LRRK2 supra-molecular complex otherwise compromised by the E193K substitution.

In conclusion, we propose that the E193K variant behaves as a partial loss of function mutation. Indeed, loss-of-function mutations have normally a recessive inheritance, while LRRK2 mutations, despite an incomplete penetrance, are well recognized as dominant^32^. Leandrou and colleagues recently characterized from a biochemical standpoint LRRK2 dimers encompassing wild type/wild type, wild type/mutant or mutant/mutant proteins^33^. Intriguingly, they found that hetero dimers of WT and mutant LRRK2 behave as wild type homodimers, suggesting that a key event in disease pathogenesis may be the gradual formation of homodimeric mutant LRRK2. In the light of this scenario, the age-dependent accumulation of dysfunctional LRRK2 E193K homodimers might affect synaptic transmission as well as mitochondrial dynamics^18^ in a dominant negative fashion. Indeed, we reported here that the G2019S variant elicits a similar increase in SV trafficking. These results are in agreement with our previous observations^11,14^ and suggest that different mutations may impinge on the control of SV cycle to execute LRRK2 pathological effect. Indeed it will be important to characterize the functional impact of E193K variant in a mammalian neuronal context.

## Conclusions

Our findings show that missense mutations outside the LRRK2 enzymatic core influence the LRRK2 supra-molecular organization and thus its physiological activity. We describe the E193K variant as a partial loss of function mutation. Together with previous reports on the G2385R mutation^4,34^, this work suggests that the pathophysiology underlying this LRRK2-associated PD mutation might be more complex than a straight gain-of-function mechanism.

## Methods

### Plasmids, cell cultures and transfection

Human LRRK2 full-length, LRRK2 914-end (hereinafter termed as LRRK2ΔN-terminus) and LRRK2 1–983 (hereinafter termed as LRRK2 N-terminus) were inserted into the pDEST57 vector (N-terminal red fluorescent protein RFP tag, Invitrogen). hLRRK2 full-length and LRRK2ΔN-terminus were inserted in N-terminal Strep-FLAG (SF-TAP) plasmid^35^ using the Gateway system (Invitrogen). Full-length RFP and Strep-FLAG E193K variants were created *via* site-directed mutagenesis using the QuikChange mutagenesis kit (Stratagene). SynaptopHluorin expressing vector was previously described^36^. N2A (Neuro2a, ATCC CCL-131) cells were grown in DMEM supplemented with 10% FBS, 1% penicillin/streptomicin and 1% glutamine in a humidified atmosphere of 5% CO_2_ at 37 °C. Cortical cultures were obtained from embryonic day 15.5–16.5 mouse as described^37,38^. N2A cells and DIV4 neurons were transfected with the different constructs using Lipofectamine 2000 (Invitrogen).

### Co-immunoprecipitation

LRRK2 pull-down from N2A cells was performed as already described^4^. In brief, 48 h after transfection cellular proteins were extracted in lysis buffer (150 mM NaCl, 2 mM EDTA, 50 mM Tris-HCl, 1% NP-40 and 0.25% sodium deoxycholate, pH 7.4) supplemented with protease and phosphatase inhibitors (Calbiochem) for 1 h at 4 °C. Samples were incubated on Strep-Tactin Superflow resin (Iba-Lifesciences) for 2 h at 4 °C. Resin was then incubated with a washing buffer (300 mM NaCl, 50 mM Tris-HCl pH 7.4). Interacting proteins were eluted in Laemmli buffer 2X at 55 °C for 10 minutes. Samples analyses by Western-blotting was performed on 10% SDS-PAGE gels. Protein samples were transferred into nitrocellulose membrane (Amersham) at 82 V for 2 h at 4 °C. The primary antibodies were applied overnight at 4 °C in blocking buffer (20 mM Tris, 150 mM NaCl, pH 7.4, 0.1% Tween 20) with 5% non-fat dry milk. Primary antibodies included: rabbit anti-LRRK2 1:500 (MJFF2, c41-2), rabbit anti-RFP 1:250 (Abcam), rabbit anti-GFP 1:5000 (Thermo Fisher), mouse anti-β-Actin 1:4000, mouse anti-α-tubulin 1:2000 (Sigma-Aldrich), rabbit anti-Synapsin I 1:1000, rabbit anti-S6 ribosomal protein 1:2000 (Cell Signaling). The secondary antibodies HRP-conjugated anti rabbit or anti mouse (Jackson Immunoresearch) were used at 1:7000 dilution. Proteins were detected using the ECL prime detection system (GE Healthcare) taking advantage of ChemiDoc Touch Imaging system (Bio-rad). Band intensity was quantified on ImageJ. To evaluate co-immunoprecipitation efficiency, the intensity of the co-immunoprecipitated protein has been normalized to the amount of LRRK2 variant immunoprecipitated.

### Immunofluorescence

DIV 14 neurons were fixed with 4% paraformaldehyde and 4% sucrose (10 minutes, room temperature). Cover slips were mounted with prolonged reagent (Life Technologies) and observed with Zeiss Observer Z1 microscope. Images were acquire using a plan-Apochromat 40x objective and pixel size 0,102 μm × 0,102 μm. Neuritic tree analysis was performed using NeuronJ.

### Synaptic vesicle isolation and binding assay

SV were isolated from rat brain via controlled-pore glass chromatography as previously described^39^. RFP-LRRK2 WT and E193K proteins were purified from transfected cells using the RFP-Trap_A kit according to manufacturer’s protocol. To appreciate LRRK2 fusion proteins binding to SV we performed a high-speed sedimentation assay^40^. Briefly, we incubated SV (5–10 µg total protein) with RFP-LRRK2 WT or RFP-LRRK2 E193K protein for 1 h at 0 °C in dedicated buffer (220 mM glycine, 30 mM NaCl, 5 mM Tris/HCl, 4 mM Hepes, pH 7.4, 0.22 mM NaN_3_, 0.2 mM PMSF, 2 μg/ml pepstatin and 100 µg/mL of bovine serum albumin). Next, we isolated LRRK2 bound to SV by high-speed centrifugation (400,000 × g for 45 min). Pellets were resuspended in Laemmli buffer 2X and analysed by western blotting with RFP antibodies. The fraction of SV bound LRRK2 in comparison to known amount of fusion proteins was normalized on SV yield, determined by western blotting with anti-synaptophysin 1:1000 (Sigma-Aldrich).

### Time lapse microscopy by total internal reflection fluorescence microscopy (TIRFM)

We imaged transfected cells 48 h after transfection via TIRF microscopy as in^20^. The microscope (Carl Zeiss Inc.) was equipped with an Argon laser at 25◦C using a 100× 1.45 numerical aperture (NA) oil immersion objective. Green fluorescence was excited with 488-nm laser line and imaged through a band-pass filter (Zeiss) onto a Retiga SRV CCD camera. Single-cell imaging under TIRF illumination was performed at 1hz for a total of thirty seconds, in a standard KRH solution (n mM: 125 NaCl, 5 KCl, 2 CaCl2, 1.2 MgSO4, 1.2 KH2PO4, 5 Glucose, 25 4-(2-Hydroxyethyl)-piperazine-1-ethanesulfonic acid (HEPES) (buffered to pH 7.4) at room temperature (25 °C). On each coverslip, up to ten cells were imaged in not less than three independent experiments for each construct. Image-Pro Plus Analyser Image Software (Media Cybernetics, Bethesda, MD, USA) was used to analyze TIRF images. A set of automated image processing macro/subroutines based on existing algorithms of the Image-Pro Plus software (High Pass Gaussian filtering, nearest neighbouring deconvolution) has been developed and corrected for photobleaching. The corrected images were subsequently analyzed by means of the Image-Pro Plus plug-in software (tracking object) that allow the selection and quantification of fluorescent spots on the basis of their shapes, size and intensity. We selected the following criteria to include individual structures in the analysis: (1) mean area 0.02–1 μm^2^, (2) minimal pixel intensity six fold over the average cell fluorescence intensity, (3) aspect (major/minor axis) 1–3, (4) velocity limit search radius 1 (micron/frame). Spots that showed up in the same position in at least 6 frames of the movie were automatically excluded from the analysis. Data were then exported in Excel for further analysis. The fluorescence intensity (F) of each spot in the various frames was normalized to the minimal pixel intensity (F0, calculated as defined above) and plotted against time. A custom written macro has been used to automatically count the number of fusion events and the whole cell fluorescence changes. Data collected from at least 20 cells for each construct were normalized to the cell area. To quantify the vesicle density in the TIRF zone, the cells were incubated for 5 minutes with 50 mM NH_4_Cl_2_ in KRH solution, to label all synapto-pHluorin positive vesicles. Then, cells were fixed in paraformaldehyde and imaged by TIRFM or epifluorescence. The number of vesicles was quantified as described above and normalized for the cell area.

### Statistical analysis and guidelines

All data included are expressed as mean ± standard error of the mean (SE). Data set was analysed *via* unpaired Student’s t test (two classes) or ANOVA followed by Tukey’s post-hoc test (more than two classes). Number of experiment (n) and level of significance (p) are indicated throughout the text. All methods were performed in accordance with the relevant guidelines and national regulations. All procedures involving animals were approved by Institutional and National Agencies (autorizzazione 793/2016-PR and 365 D.lgs 116/92-art.7, IRCCS San Martino-IST, PROT. n. 0005278/14).

## Supplementary information

Supplementary material.
