# Supplementary material for: The LRRK2 N-terminal domain influences vesicle trafficking: impact of the E193K variant
Source: Sci Rep. 2020 Mar 2;10:3799. doi: 10.1038/s41598-020-60834-5 (PMC7052203; doi:10.1038/s41598-020-60834-5)
Supplement: Supplementary file 1 — Supplementary material. [file 41598_2020_60834_MOESM1_ESM.pdf]

The LRRK2 N-terminal domain influences vesicle trafficking: impact of the E193K variant

Algerta Marku[#], Maria Dolores Perez Carrion[#], Francesca Pischedda, Antonella Marte, Zeila Casiraghi, Paola Marciani, Felix von Zweydorf, Christian Johannes Gloeckner, Franco Onofri, Carla Perego[*], Giovanni Piccoli[*]

Supplementary Figure 1. (A-D) The graphs show RFP-LRRK2 derived construct (A,C) and sypHy (B,D) optical density normalized upon S6 ribosomal protein (S6RP) level as detected by western-blotting. Data are expressed as mean ± SE n=5. (E-F) The graph shows the average peak height of the synaptic events for the different constructs. Data are expressed as mean ± SE of up to 20 cells per construct; *p<0.05 vs E193K.

Supplementary Figure 2. G2019S variant affects vesicle trafficking. (A) Schematic representation of LRRK2 wild-type and G2019S variant. The distinct LRRK2 domains are indicated. (B) Western-blotting analysis of cells expressing synaptopHluorin reporter (sypHy) together with RFP-LRRK2 derived constructs. (C-D) The graphs show RFP-LRRK2 derived construct (C) and sypHy (D) optical density normalized upon S6 ribosomal protein (S6RP) level as detected by western-blotting. Data are expressed as mean ± SE n=6. (E) Time course analysis of synaptic events occurring in N2A cells expressing LRRK2 wild-type (WT) or G2019S LRRK2 and the sypHy reporter. Transfected cells were imaged by TIRFM 48h later. Peaks of variable fluorescence intensity correspond to single fusion events. Fluorescence data are expressed as F/F0. The graphs show the total number of fusion events (F), the resulting fluorescence changes (G) expressed as Area Under Curve (AUC), and the average peak height of the synaptic events for each construct. Data are normalized for the cell area and are expressed as mean ± SE of up to 32 cells per construct in five independent experiments; **p<0.01, ***p<0.001, Student's T-test.

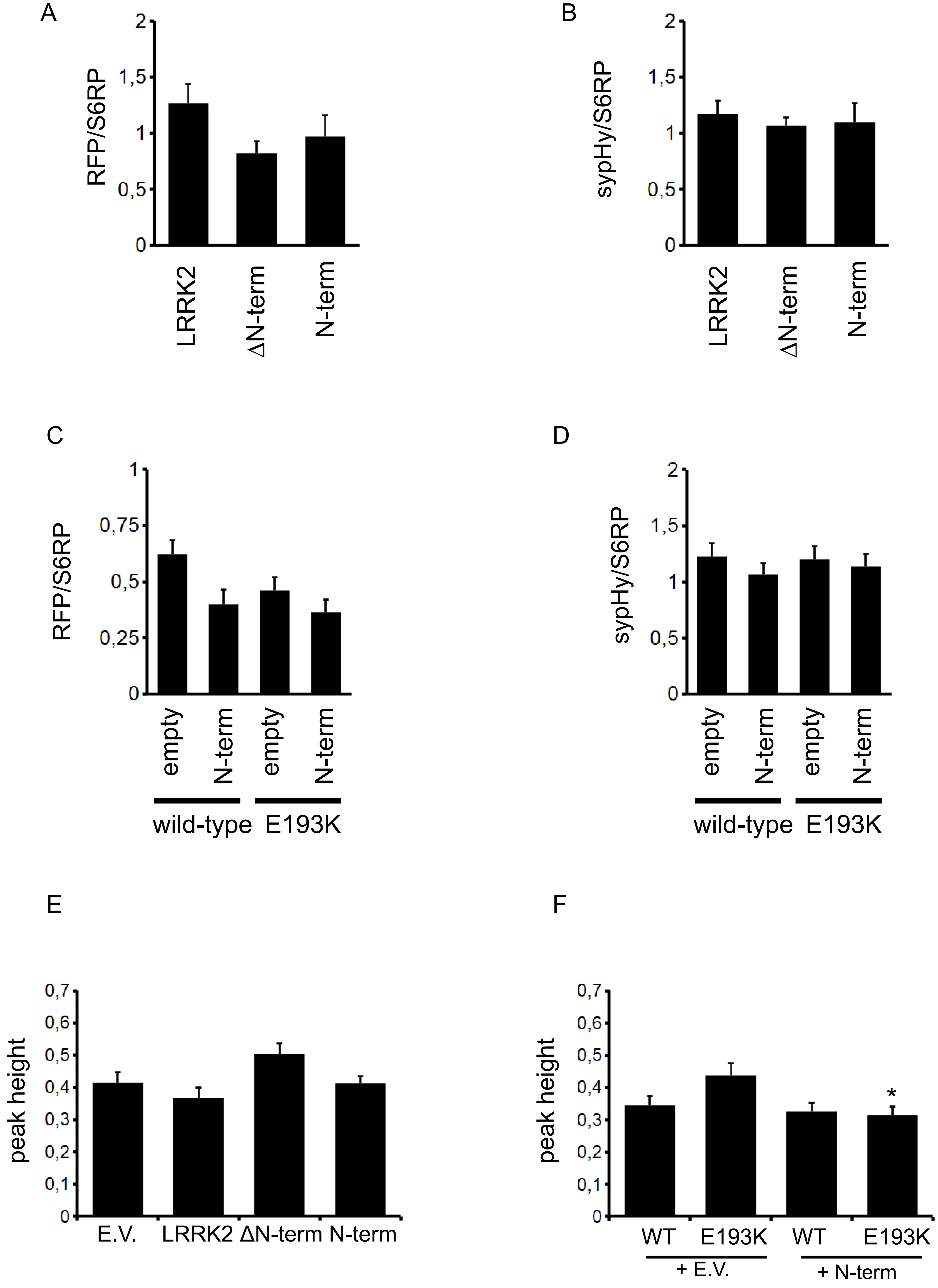

Supplementary Figure 1

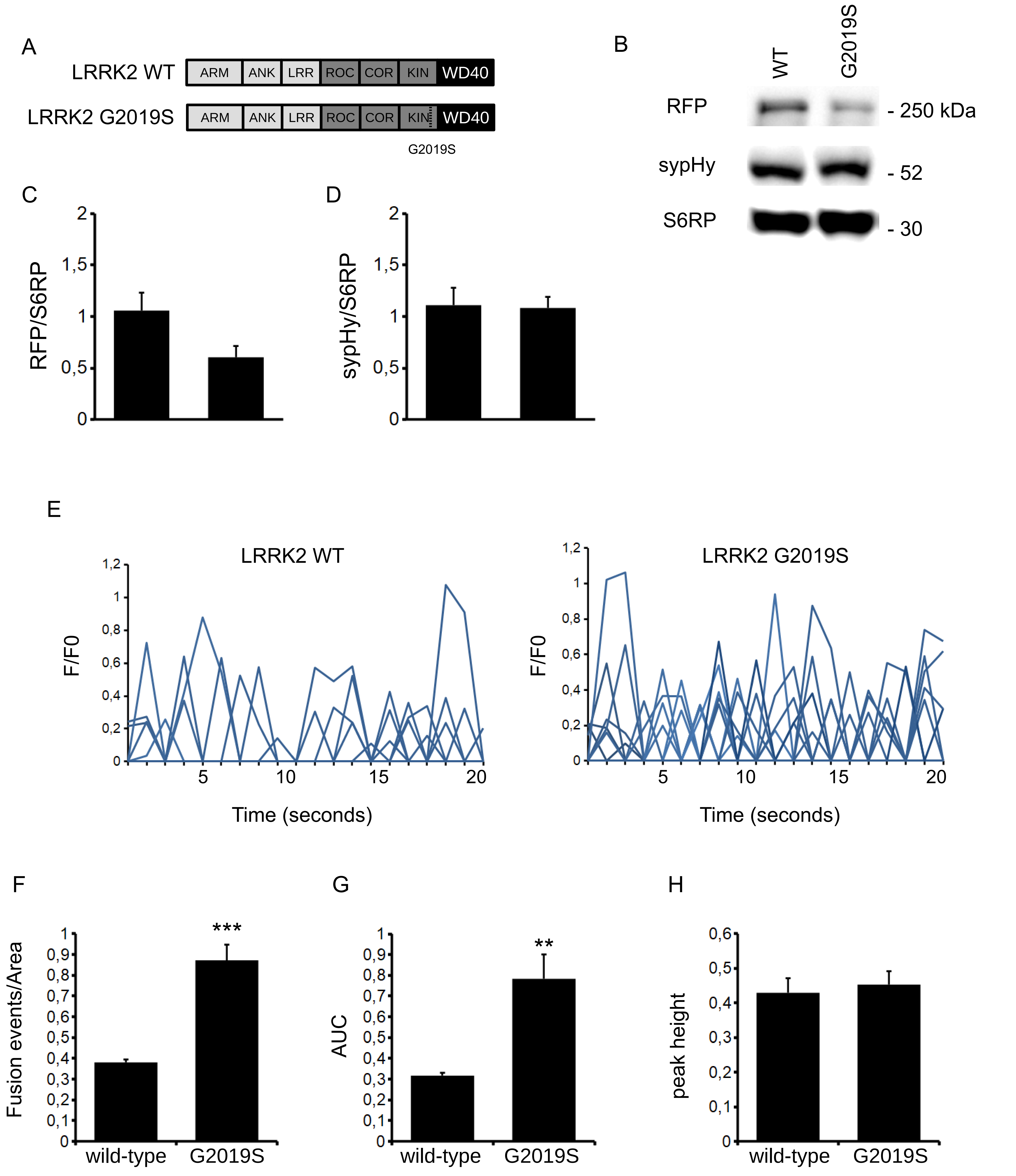

Supplementary Figure 2
